# The immunometabolite itaconate stimulates OXGR1 to promote mucociliary clearance during the pulmonary innate immune response

**DOI:** 10.1172/JCI160463

**Published:** 2023-03-15

**Authors:** Yi-Rong Zeng, Jun-Bin Song, Dezheng Wang, Zi-Xuan Huang, Cheng Zhang, Yi-Ping Sun, Gang Shu, Yue Xiong, Kun-Liang Guan, Dan Ye, Pu Wang

**Affiliations:** 1 Shanghai Key Laboratory of Clinical Geriatric Medicine, Huadong Hospital, Fudan University, and Key Laboratory of Metabolism and Molecular Medicine (Ministry of Education), and Shanghai Key Laboratory of Medical Epigenetics, International Co-laboratory of Medical Epigenetics and Metabolism (Ministry of Science and Technology), and Molecular and Cell Biology Lab, Institutes of Biomedical Sciences, Shanghai Medical College of Fudan University, Shanghai, China.; 2 College of Animal Science, South China Agricultural University, Guangzhou, China.; 3 Cullgen Inc., San Diego, California, USA.; 4 Department of Pharmacology and Moores Cancer Center, UCSD, La Jolla, California, USA.

**Keywords:** Metabolism, G protein–coupled receptors

## Abstract

Pathogens and inflammatory conditions rapidly induce the expression of immune-responsive gene 1 (*IRG1*) in cells of myeloid lineage. *IRG1* encodes an aconitate decarboxylase (ACOD1) that produces the immunomodulatory metabolite itaconate (ITA). In addition to rapid intracellular accumulation, ITA is also secreted from the cell, but whether secreted ITA functions as a signaling molecule is unclear. Here, we identified ITA as an orthosteric agonist of the GPCR OXGR1, with an EC_50_ of approximately 0.3 mM, which was in the same range as the physiological concentration of extracellular ITA upon macrophage activation. ITA activated OXGR1 to induce Ca^2+^ mobilization, ERK phosphorylation, and endocytosis of the receptor. In a mouse model of pulmonary infection with bacterial *Pseudomonas aeruginosa*, ITA stimulated Oxgr1-dependent mucus secretion and transport in respiratory epithelium, the primary innate defense mechanism of the airway. Our study thus identifies ITA as a bona fide ligand for OXGR1 and the ITA/OXGR1 paracrine signaling pathway during the pulmonary innate immune response.

## Graphical abstract

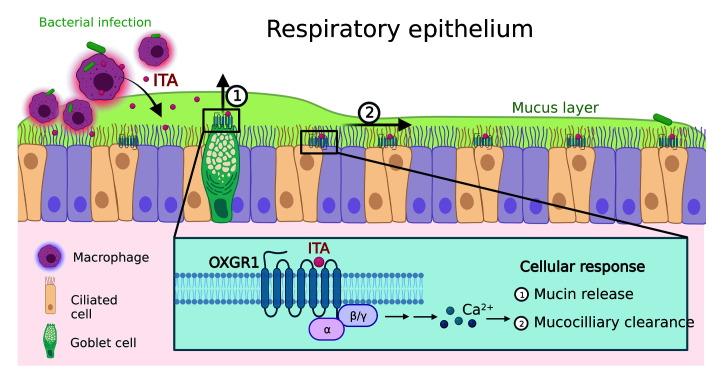

## Introduction

Metabolic reprogramming is a fundamental mechanism for regulating the immune response in both innate and adaptive immune systems (1). Itaconate (ITA), a dicarboxylic acid, is one of the best examples of metabolic reprogramming during the immune response (2). ITA has long been known as one of the major building-block chemicals that can be produced from sugars via biological conversions in fungi *Aspergillus* for the industrial-scale synthesis of a number of high-value, bio-based chemicals or materials (3). ITA was also discovered in activated macrophages of high eukaryotes (4). The enzyme that produces ITA has been identified as the mitochondrially localized aconitate decarboxylase (ACOD1) (5), encoded by immune-responsive gene 1 (*IRG1*, NCBI Gene ID: 730249), which was first identified by its rapid transcriptional induction in macrophages challenged by bacterial LPS (6). The expression of IRG1/ACOD1 is restricted to cells of myeloid lineages, in particular in activated macrophages that play major roles in host defense against various microbial pathogens such as bacteria and fungi, supporting the function of ITA in the innate immune defense.

Several mechanisms have been proposed for the immunomodulatory function of ITA. These include the inhibition of succinate dehydrogenase (SDH), which results in succinate accumulation and metabolic reprogramming (7, 8), and alkylation of protein cysteine residues, which induces the electrophilic stress response mediated by NRF2 and IκBζ (9, 10) and impairs aerobic glycolysis (11). We recently discovered that ITA, which is structurally similar to the Krebs cycle intermediate α-ketoglutaric acid (α-KG), binds to and inhibits α-KG–dependent TET DNA demethylases and thereby downregulates NF-κB and STAT target genes to dampen the inflammatory response (12). Intriguingly, ITA was also noted to secrete from activated macrophages into the inflamed microenvironment (4, 8). Whether secreted ITA plays a role in mediating the function of IRG1 in modulating the immune response remains largely unknown.

Macrophages in the lungs are the first responders to pulmonary bacterial and fungal pathogens, including bacterial *Pseudomonas aeruginosa* (*P*. *aeruginosa*), an opportunistic pathogen known to infect patients during existing diseases or conditions such as cystic fibrosis (CF) and also known for its multiple drug resistance (13). The pulmonary respiratory epithelium acts as a physical barrier to airborne pathogens through a self-cleaning mechanism called mucociliary clearance (MCC) (14). Goblet cells and other cells secret a mucus layer that covers the respiratory epithelium to trap inhaled particles, and the cilia beating of ciliated cells continuously transports the mucus into the pharynx. Dysfunction of MCC is associated with multiple diseases such as CF, asthma, and chronic obstructive pulmonary disease (COPD) (14). Nucleosides sensed by purinergic receptors are major regulators of cilia beating and mucin secretion (15), although many other signals are also important, such as paracrine signaling from innate immune cells during infection (16). Of note, ITA was detected in mouse airways after pathogen infection (17, 18), and its concentration was increased in sputum samples from patients with CF (19). *Irg1*-KO mice were more vulnerable to pulmonary infection with diverse pathogens, such as *Mycobacterium tuberculosis*, *Brucella*, *Legionella*, influenza A virus, respiratory syncytial virus, and so on (20–24). *P. aeruginosa* and *Staphylococcus aureus* can exploit host-derived ITA to fuel biofilm formation and thus protect them from clearance by immune defense systems (18, 19). Together, these findings suggest that airway-secreted ITA is a conserved feature that influences the evolution of many airborne pathogens.

Multiple GPCRs play an important role in sensing metabolites to regulate immune function (25, 26). For instance, *OXGR1* (also called *GPR99,* NCBI Gene ID: 27199) encodes a rhodopsin family GPCR and was reported to be stimulated by α-KG (27). OXGR1 is expressed in the respiratory epithelium and is indispensable for MCC (28). In the studies of respiratory tract infection by *Alternaria*, deletions of *Oxgr1* in mice were reported to cause decreased mucin secretion and brush cell expansion (28, 29). *Oxgr1-*null mice also spontaneously developed otitis media that was probably caused by impaired airway epithelium function in the eustachian tube (30). Renal α-KG/OXGR1 signaling was found to promote bicarbonate secretion and maintain the acid-base balance of body fluids (31). Despite being highly concentrated in urine (32), α-KG levels in serum and other body fluids are far lower than its EC_50_ for OXGR1 (33), suggesting that α-KG is not likely to be the physiological ligand for OXGR1 in the respiratory epithelium or most other tissues. In this study, we identified ITA as an orthosteric agonist of OXGR1 and determined that the ITA/OXGR1 signaling pathway plays an important role in the self-cleaning function of the airway during the pulmonary innate immune response.

## Results

### A screen of GPCR receptors discovers OXGR1 as an ITA receptor.

**Figure 1:**
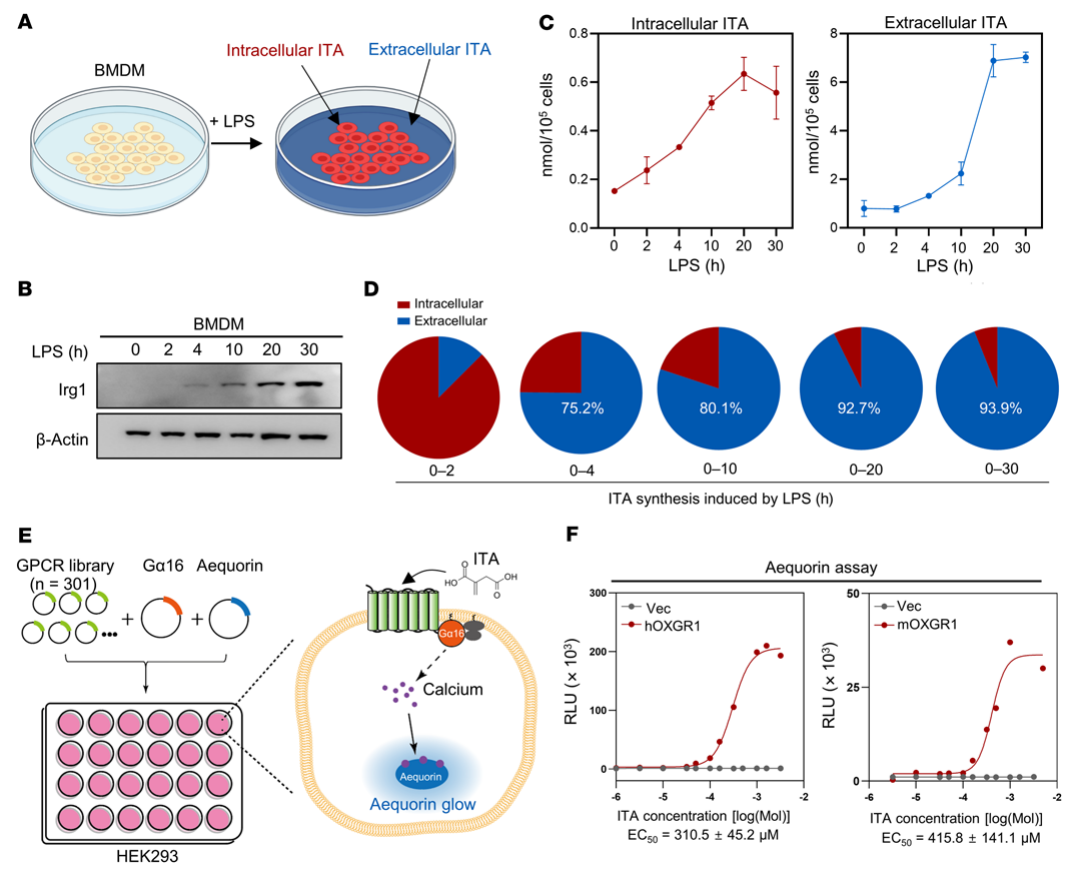
A screen of GPCR receptors discovers OXGR1 as a potential ITA receptor. (**A**) In mouse BMDMs challenged with LPS, intracellular and extracellular levels of ITA were collected and quantified. (**B**) Immunoblot of Irg1 in LPS-challenged mouse BMDMs. (**C**) Intracellular and extracellular levels of ITA were measured by LC-MS in LPS-challenged BMDMs at the indicated time points. Error bar, SEM. (**D**) Comparison of the percentage of newly synthesized intracellular and extracellular ITA during LPS stimulation. (**E**) Schematic illustration of the GPCR screen. A total of 301 plasmids encoding for GPCRs were transfected along with G_α16_ and aequorin plasmids. ITA-induced aequorin luminescence was determined. (**F**) The ITA dose response was determined by the aequorin assay in HEK293 cells overexpressing human OXGR1 (hOXGR1) or mouse OXGR1 (mOXGR1). Vec, empty vector.

To gain insight into the function of secreted ITA, we challenged mouse bone marrow–derived macrophages (BMDMs) with LPS and found, as expected, that the induction of Irg1 protein led to increased levels of both intracellular and extracellular ITA (Figure 1, A–C). Notably, a substantial amount of ITA was secreted into the culture medium starting at 4 hours after LPS treatment, and the extracellular ITA continuously accumulated and eventually comprised more than 90% of the newly synthesized ITA (Figure 1D). The absolute concentration of extracellular ITA was 48.9 ± 0.6 μM twenty hours after LPS treatment at a routine plating density (8 × 10^5^ BMDMs in 1 mL medium per well) (Figure 1C).

In our search for the function and mechanism of secreted ITA, we noted the signaling of the GPCR proteins SUCNR1/GPR91 and OXGR1/GPR99, which sense the TCA metabolites succinate and α-KG, which are structurally similar to ITA (27). To test the hypothesis that ITA may similarly signal through a GPCR, we performed a screening of human GPCRs in transfected HEK293 cells and measured GPCR-dependent calcium mobilization (Figure 1E and Supplemental Figure 1; supplemental material available online with this article; https://doi.org/10.1172/JCI160463DS1). In order to allow more GPCRs to couple with the calcium pathway, we optimized the screening system by inducing coexpression of the promiscuous G_q_ family α subunit Gα16 (34). We examined 301 GPCRs that cover more than 70% of the nonolfactory GPCRs encoded by the human genome (35). Strikingly, we found that OXGR1 was the only receptor that showed robust and reproducible calcium mobilization in response to ITA treatment (Supplemental Figure 1).

The EC_50_ of ITA for human OXGR1 was 200–300 μM, which was comparable to the EC_50_ of α-KG (Figure 1F and Supplemental Figure 2, A–C). The EC_50_ of ITA for mouse OXGR1 was approximately 400 μM, and the EC_50_ of α-KG was in the same range (Figure 1F and Supplemental Figure 2D). Considering that secreted ITA would be greatly diluted in the large volume of culture medium, the concentration of extracellular ITA around activated macrophages in vivo could be higher than the EC_50_ of ITA for OXGR1 and could thus activate this potential receptor.

### ITA acts as an agonist of OXGR1 to stimulate cytosolic calcium, ERK activation, and receptor endocytosis.

**Figure 2:**
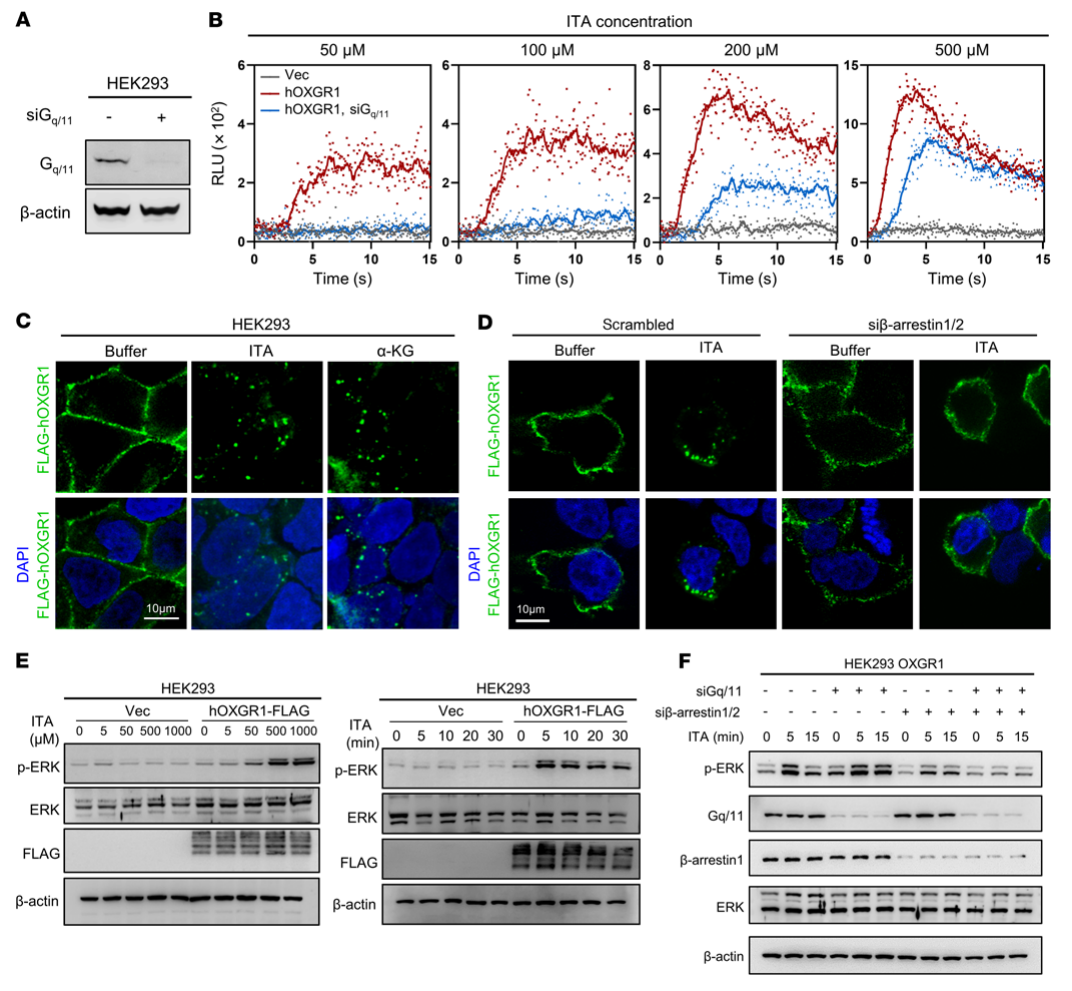
ITA acts as an agonist of OXGR1 to stimulate cytosolic calcium, ERK activation, and receptor endocytosis. (**A**) Verification of G_q/11_ knockdown in HEK293 cells by Western blotting. (**B**) Aequorin assay showing calcium mobilization by different concentrations of ITA in cells expressing human OXGR1 with or without siRNAs targeting G_q/11_. (**C**) ITA and α-KG induced endocytosis of FLAG-tagged OXGR1, which was detected using anti-FLAG antibody. Nuclei were stained with DAPI. Scale bar: 10 μm. (**D**) Endocytosis of FLAG-tagged OXGR1 induced by ITA with or without siRNAs targeting β-arrestin 1/2 was detected by anti-FLAG antibody. Nuclei were stained with DAPI. Scale bar: 10 μm. (**E**) Serum-starved cells were stimulated with different concentrations of ITA (from 5 μM to 1 mM) for 15 minutes (left), or 500 μM ITA for different durations (from 5 to 30 minutes) (right). Cells were harvested, and p-ERK was determined by Western blotting. (**F**) Serum-starved cells expressing human OXGR1 transfected with siRNAs targeting G_q/11_ and β-arrestin 1/2 were stimulated with 500 μM ITA for the indicated durations. Cells were harvested, and p-ERK levels were determined by Western blotting.

Heterotrimeric G proteins mainly relay the signals from GPCRs to intracellular signal transduction (36). To investigate the intracellular signaling pathways initiated by ITA/OXGR1, we measured and found that ITA stimulated the calcium response in a dose-dependent manner in HEK293 cells overexpressing human OXGR1 and that depletion of G_q/11_ by siRNAs greatly attenuated the cellular calcium response initiated by ITA/OXGR1 (Figure 2, A and B). In contrast, ITA/OXGR1 did not affect intracellular cAMP, which is known to be influenced by G_s_ or G_i_ (Supplemental Figure 3, A and B). ITA/OXGR1 also failed to affect stress fiber formation, which is a typical response to G_12/13_ activation (Supplemental Figure 3C). These findings thus suggest that ITA/OXGR1 couples to G_q/11_, but not G_s_, G_i_, or G_12/13_ family G proteins.

Initiation of GPCR signaling by ligand binding is followed by β-arrestin–induced receptor endocytosis, which is a major mechanism for GPCR signaling termination (37). We found that ectopically expressed OXGR1 was localized on the plasma membrane and that rapid endocytosis of OXGR1 was triggered by ITA as well as α-KG within 10 minutes of ligand treatment (Figure 2C). Ablation of β-arrestin 1/2 by siRNAs impaired ITA-induced endocytosis of OXGR1 (Figure 2D), confirming that the internalization of OXGR1 was dependent on β-arrestin. Phosphorylation of ERK (p-ERK) commonly occurs in cells with activated calcium and/or β-arrestin pathways (36, 37). As expected, the phosphorylation of ERK was dose-dependently induced by ITA in OXGR1-expressing cells, and such induction was observed as quickly as 5 minutes after ITA treatment (Figure 2E). Codepletion of β-arrestin and G_q/11_, but not either single depletion alone, abolished ITA-induced ERK phosphorylation in OXGR1-expressing HEK293 cells (Figure 2F), indicating that extracellular ITA stimulates OXGR1 to initiate intracellular signaling through G_q_ and β-arrestin.

### ITA activates OXGR1 by interacting with its orthosteric site.

**Figure 3:**
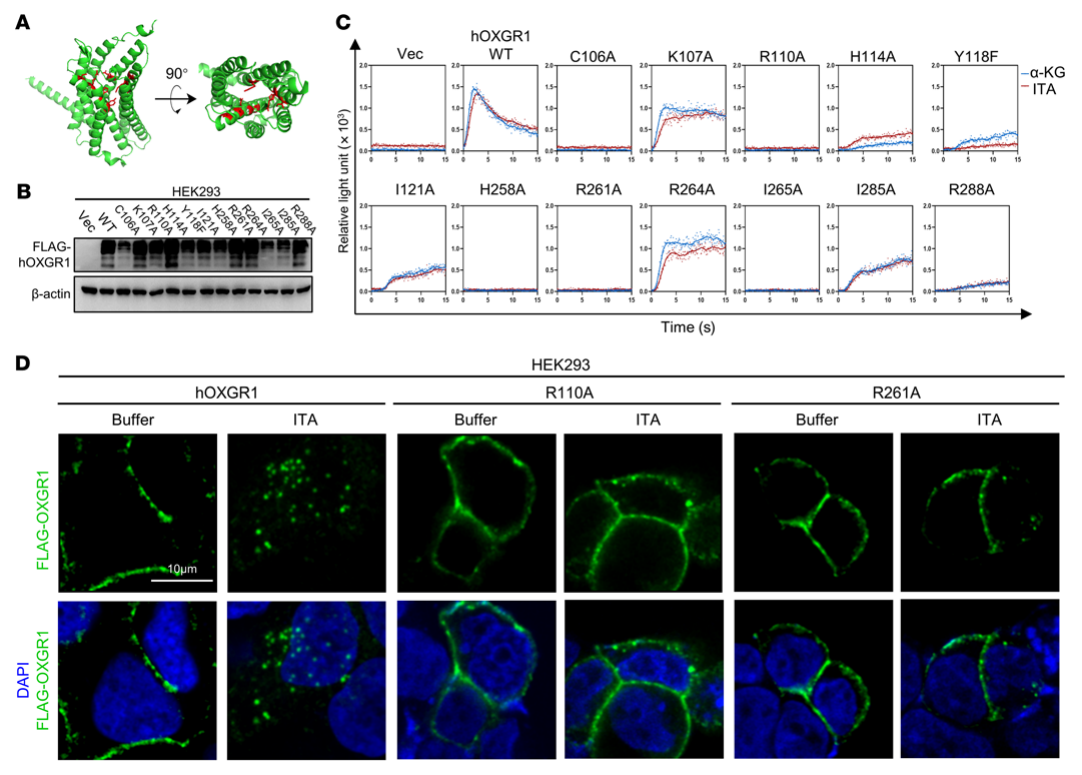
ITA activates OXGR1 by interacting with its orthosteric site. (**A**) Virtual structure of human OXGR1 predicted by AlphaFold. Amino acid residues C106, R110, H114, Y118, H258, R261, I265, and R288 are labeled in red. (**B**) Verification of ectopically expressed OXGR1 in HEK293 cells as determined by Western blotting. (**C**) Aequorin assay showing calcium mobilization in cells expressing WT or mutant OXGR1 in response to the indicated ligands. (**D**) Endocytosis of FLAG-tagged WT or mutant OXGR1 was detected using anti-FLAG antibody. Nuclei were stained with DAPI. Scale bar: 10 μm.

Most natural GPCR agonists interact with the orthosteric site of the rhodopsin family GPCRs to induce a conformational change and the guanine nucleotide exchange factor activity of GPCR. To gain additional evidence supporting ITA/OXGR1 signaling and mechanistic insights into ITA-induced OXGR1 activation, we mutated several amino acid residues localized around the orthosteric site of human OXGR1 that are predicted by the AlphaFold structure (38). These mutants are predicted to localize on the transmembrane domains TM3 (C106A, K107A, R110A, H114A, Y118F, I121A), TM6 (H258A, R261A, R264A, I265A), and TM7 (I285A and R288A) (Figure 3A). Among the mutants examined, the H114A, Y118F, I121A, I285A, or R288A mutant of OXGR1 dramatically impaired the effect of ITA or α-KG on stimulation of the calcium response, and the C106A, R110A, H258A, R261A, or I265A mutant completely abolished this response (Figure 3, B and C). Furthermore, the R110A or R261A mutant of OXGR1 did not undergo endocytosis after ITA treatment (Figure 3D). We then conducted a cellular thermal shift assay and found that ITA increased the thermal stability of WT OXGR1, but not of the R110A mutant, which was unresponsive to ITA in a calcium assay. As a negative control, the thermal stability of the cotransfected BirA protein remained unchanged (Supplemental Figure 4). Our data thus support the notion that ITA activates OXGR1 by interacting with its orthosteric site.

### ITA accumulates in the respiratory epithelium during bacterial infection.

A previous study reported that Oxgr1 was expressed in the respiratory epithelium and that *Oxgr1^–/–^* mice had impaired respiratory function (28), but the natural ligand of Oxgr1 in the respiratory epithelium has not been defined. Leukotriene E4 (LTE4) is an immunometabolite produced by mast cells and was previously reported to act as a ligand of Oxgr1 (28, 29, 39). As expected, an aequorin assay revealed that LTE4 could activate its well-known receptor CysLTR1 in cells (Supplemental Figure 5A). In contrast, LTE4 failed to show any agonizing effect on OXGR1 in both the aequorin and endocytosis assays (Supplemental Figure 5, A and C). Moreover, addition of LTE4 did not prevent, and even moderately enhanced, α-KG– or ITA-triggered intracellular Ca^2+^ release in OXGR1-expressing HEK293 cells (Supplemental Figure 5B). These results thus indicate that LTE4 was not a direct agonist of OXGR1 in our experimental system.

We next determined the in vivo concentrations of ITA and α-KG in bronchoalveolar lavage (BAL) in the mouse model of pulmonary infection with the *Pseudomonas aeruginosa* strain PAO1. Our liquid chromatography–MS (LC-MS) results determined the absolute concentration of ITA to be 0–0.067 nM and 122–689 nM in BAL isolated from uninfected and PAO1-infected mice, respectively (Supplemental Figure 6, A and B). We also estimated the ITA concentration in undiluted epithelial lining fluid (ELF) using the urea concentration in BAL and plasma as a marker of dilution (ITA_[ELF]_ = ITA_[BAL]_ × Urea _[plasma]_ / Urea _[BAL]_) (40). The ITA concentration in undiluted ELF from PAO1-infected mice was 109.5 ± 69.2 μM (ranging from 18–215 μM), indicating that endogenous ITA could accumulate to a concentration that could robustly activate OXGR1 (Supplemental Figure 6C). KO of Irg1, but not Oxgr1, abolished ITA secretion into the BAL (Supplemental Figure 6D). In contrast, gas chromatography–MS (GC-MS) analysis revealed that the detection threshold for α-KG was approximately 125 nM, but the concentration of α-KG in all BAL samples examined was under this threshold, even after the BAL samples from 4 mice were pooled and concentrated into 20 μL (Supplemental Figure 6, E–G). We thus estimated the α-KG concentration in undiluted epithelial lining fluid to be below 40.7 nM using an average dilution factor determined by the urea concentration, which was too low to activate Oxgr1 (EC_50_ of α-KG is 241.1 ± 61.1 μM for mouse Oxgr1).

Taken together, these results suggest that ITA, but not LTE4 or α-KG, is the natural ligand of Oxgr1 in respiratory epithelium during bacterial infection.

### ITA signals through OXGR1 to stimulate MCC in respiratory epithelium.

**Figure 4:**
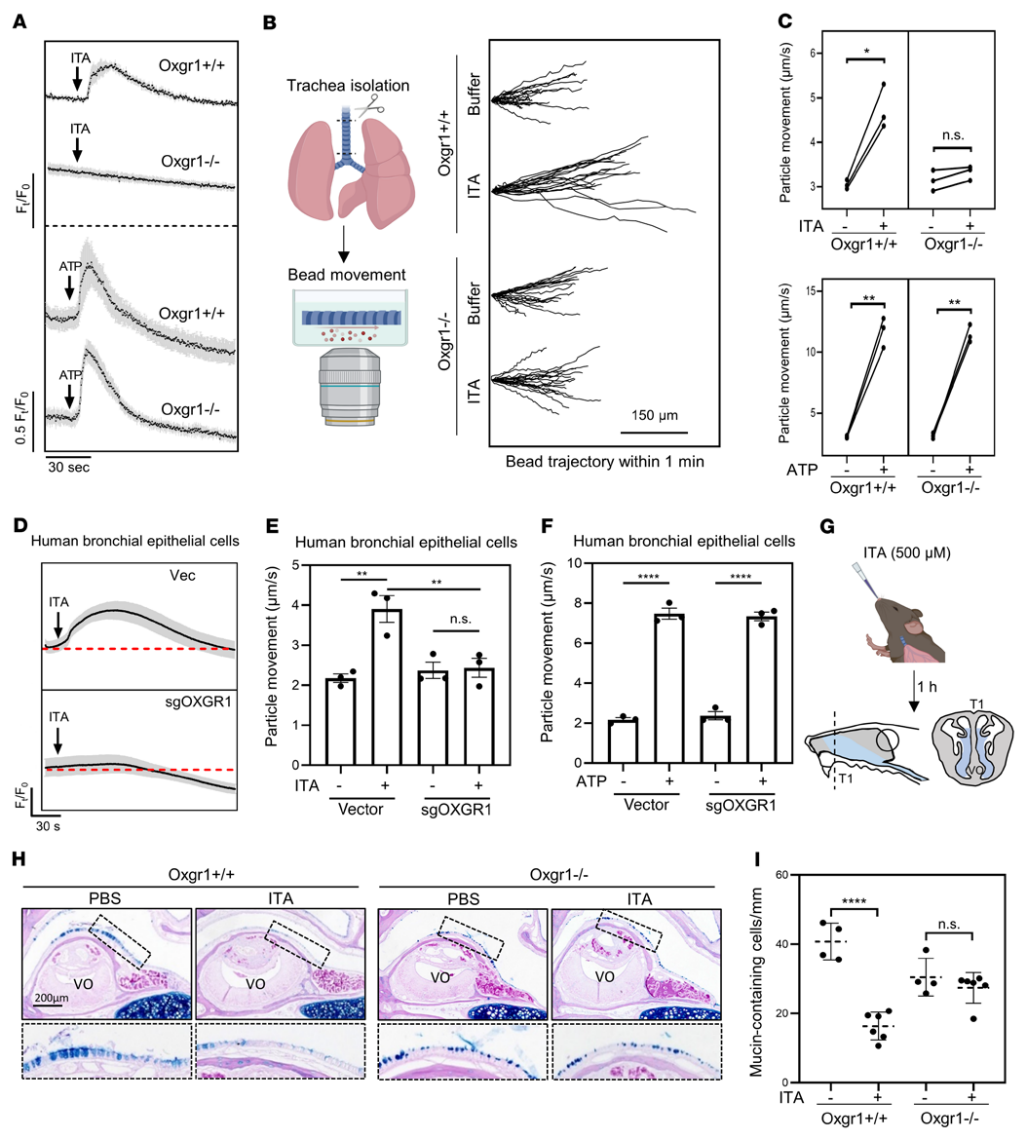
ITA signals through OXGR1 to stimulate MCC in respiratory epithelium. (**A**) Calcium mobilization of primary trachea epithelial cells isolated from mice in response to 670 μM ITA or 100 μM ATP, as determined by Fluo-4AM assay. (**B**) Tracheas were cultured ex vivo, and cilia beating–derived fluid flow was monitored by tracing fluorescent bead movement under the microscope. The trajectory of 20 individual beads in 1 minute with or without 500 μM ITA is shown. Scale bar: 150 μm. (**C**) Quantification of bead movement speed in trachea culture from individual *Oxgr1^+/+^* (*n* = 3) and *Oxgr1^–/–^* (*n* = 3) mice in the presence of 500 μM ITA or 100 μM ATP. Each dot represents an independent biological replicate. *P < 0.05 and **P < 0.01, by paired, 2-tailed Student’s *t* test. (**D**) Primary human bronchial epithelium cells were infected by a control lentivirus (Vec) or viruses expressing sgRNAs targeting OXGR1 (sgOXGR1). Calcium mobilization in response to 500 μM ITA was determined by Fluo-4AM assay. (**E** and **F**) Comparison of bead movement speed between ALI culture of human bronchial epithelium cells in the presence of 500 μM ITA or 100 μM ATP. **P < 0.01 and ****P < 0.0001, by 1-way ANOVA (**E**) or unpaired, 2-tailed Student’s *t* test (**F**). Data indicate the mean ± SEM. (**G**–**I**) Anesthetized mice of the indicated genotype (*n* = 4–6 per group) were intranasally administered PBS or 500 μM ITA, and their nasal cavities were harvested 1 hour after ITA administration. (**G**) Sections were taken from the most proximal aspect of the nasal cavities. (**H**) Representative images of Alcian blue/PAS staining of nasal cavities are shown. (**I**) Quantification of mucin-containing cells in the indicated groups of animals. ****P < 0.0001, by 2-tailed Student’s *t* test. Data indicate the mean ± SEM.

Accumulation of ITA in the respiratory epithelium prompted us to examine the potential role of ITA/OXGR1 signaling in respiratory mucosal immunity. To this end, we isolated primary respiratory epithelial cells from *Oxgr1^+/+^* and *Oxgr1^–/–^* mice, followed by ITA treatment and detection of the cytoplasmic calcium concentration with the calcium dye Fluo-4AM. We observed that both *Oxgr1^+/+^* and *Oxgr1^–/–^* cells exhibited normal calcium release in response to ATP, which stimulates the purinergic receptors P2X and P2Y (15), suggesting that loss of Oxgr1 did not have a broadly deleterious effect on the general function of respiratory epithelial cells (Figure 4A). In line with our earlier observation made in transfected HEK293 cells (Supplemental Figure 2A), treatment with ITA led to rapid calcium mobilization in *Oxgr1^+/+^*, but not *Oxgr1^–/–^* respiratory epithelial cells (Figure 4A). Consistently, treatment of primary human bronchial epithelial cells with 0.5 mM ITA also led to robust calcium mobilization, and such ITA-stimulated calcium mobilization was abolished by depletion of OXGR1 (Figure 4D), providing evidence of the physiological relevance of ITA/OXGR1 signaling in human cells.

Calcium signaling promotes the beating of motile cilia and mucociliary transport by respiratory epithelial cells (15). To test whether ITA and OXGR1 regulate mucociliary transport, we isolated the mouse trachea and then cultured it ex vivo in a medium containing 400 nm diameter fluorescent beads to trace culture medium movement (Figure 4B). We found that ITA treatment significantly increased the movement of beads driven by trachea derived from *Oxgr1^+/+^*, but not *Oxgr1^–/–^*, mice (Figure 4, B and C), supporting the notion that ITA signals through the Oxgr1 receptor to boost mucociliary transport of the liquid. As a negative control, trachea tissues from both *Oxgr1^+/+^* and *Oxgr1^–/–^* mice exhibited comparable speed of bead movement in response to ATP (Figure 4C). In addition, we reconstructed ciliated respiratory epithelium by air-liquid interface (ALI) culturing of primary mouse respiratory epithelial cells and observed that ITA could accelerate the movement of beads in an Oxgr1-dependent manner (Supplemental Figure 7). Likewise, ITA treatment also accelerated the movement of beads in primary human respiratory epithelial cells under ALI culture in an OXGR1-dependent manner (Figure 4, E and F), reaffirming that ITA stimulates endogenous OXGR1 in human bronchial epithelial cells.

GPCR-induced calcium signaling promotes rapid mucin secretions from the respiratory epithelium (15). We found that intranasal administration of ITA for 1 hour led to rapid mucin secretion, as observed by a decrease of mucin-containing cells in the respiratory epithelium around the vomeronasal organ (VO) in *Oxgr1^+/+^* mice (Figure 4, G–I). However, we did not observe an effect of ITA on the reduction of mucin-containing cells in the respiratory epithelium of *Oxgr1^–/–^* mice, suggesting that ITA promoted *Oxgr1*-dependent mucin release in a physiologic setting.

### Depletion of either Oxgr1 or Irg1 disrupts the clearance of respiratory bacteria.

**Figure 5:**
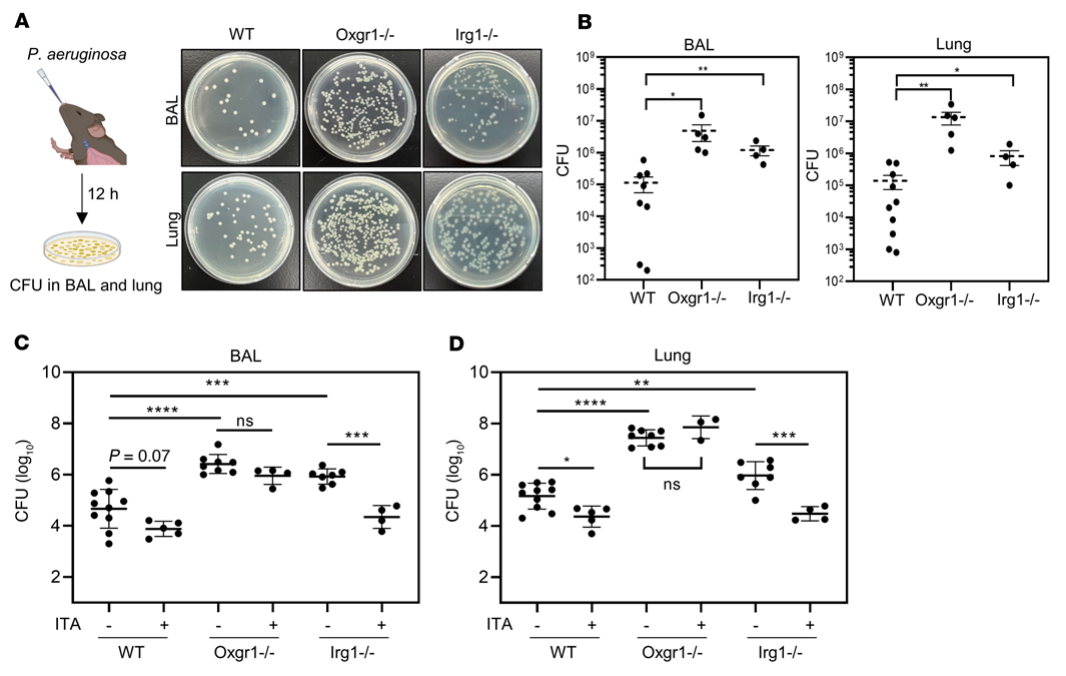
Depletion of either Oxgr1 or Irg1 disrupts the clearance of respiratory bacteria. (**A** and **B**) Anesthetized mice of the indicated genotype (*n* = 4–10 per group) were intranasally administrated the *P*. *aeruginosa* strain PAO1 (1 × 10^7^ CFU). BAL and lung tissue were collected 12 hours after infection. Representative images of PAO1 colonies on CTAB-selective plates after overnight culturing of BAL and lung from infected mice (dilution: 10^4^-fold) (**A**) and quantification of CFU (**B**) are shown. *P < 0.05 and **P < 0.01, by 1-way ANOVA. Data indicate the mean ± SEM. (**C** and **D**) Anesthetized mice of the indicated genotype (*n* = 4–10 per group) were intranasally administrated 20 μL saline containing PAO1 (1 × 10^7^ CFU) with or without 1 mM ITA. The CFU in BAL (**C**) and lung tissues (**D**) were quantified. **P < 0.01, ***P < 0.001, and ****P < 0.0001, by 1-way ANOVA. Data indicate the mean ± SEM.

MCC of inhaled pathogens is the primary innate defense mechanism of the airway. A previous study reported that ITA was readily detected in the BAL from mice infected with *P*. *aeruginosa* and that the clearance of this Gram-negative bacterium was impaired in *Irg1*-KO mice (18). To determine whether ITA/Oxgr1 signaling participates in airway pathogen clearance, WT, *Oxgr1^–/–^*, and *Irg1^–/–^* mice were subjected to intranasal infection with the *P*. *aeruginosa* strain PAO1, and BAL and lung tissue were collected 12 hours after infection to count CFU (Figure 5A). In accord with the impaired MCC observed in a previous study (18), *Irg1^–/–^* mice exhibited a remarkable increase of bacteria in BAL and lung tissue as compared with WT controls (Figure 5B). Notably, the phenotype of defective airway pathogen clearance was recapitulated in the same infection model in *Oxgr1^–/–^* mice (Figure 5B). To provide further direct evidence supporting the role of IRG1/ITA/OXGR1 signaling in the innate bacterial defense, we sought to determine whether ITA supplementation rescues the bacterial overgrowth phenotype. The dose of supplemented ITA was adjusted to reach 200–300 nM in the BAL 0.5 hours after ITA supplementation, which mimics the endogenous ITA concentration in WT mice stimulated by PAO1 infection for 12 hours, although the concentration of exogenous ITA was rapidly decreased because of self-cleaning of the respiratory epithelium (Supplemental Figure 8A). ITA was intranasally supplemented in WT, *Oxgr1^–/–^*, and *Irg1^–/–^* mice infected with *P*. *aeruginosa*. We found that ITA supplementation rescued the bacterial overgrowth phenotype in *Irg1^–/–^*, but not *Oxgr1^–/–^*, mice (Figure 5, C and D).

Mucins and antimicrobial polypeptides are essential immune defense components secreted by the respiratory tract (41, 42). To test the hypothesis that ITA/OXGR1 signaling may alter the concentration of mucins and antimicrobial polypeptides, we performed an ELISA to quantify the concentration of mucin 5b and β-defensin 1 in the mouse models of *P*. *aeruginosa* infection. Neither ITA intranasal supplementation nor Irg1 or Oxgr1 KO altered the levels of mucin 5b or β-defensin 1 in the BAL (Supplemental Figure 8, B and C). Nevertheless, we found that mucin 5b or β-defensin 1 levels were positively correlated with the ITA concentration in BAL from WT mice after *P*. *aeruginosa* infection (Supplemental Figure 8, D and E). It should be noted that Oxgr1 activation by ITA can simultaneously facilitate mucus secretion and mucus transport (Figure 4). These 2 “opposite” effects of ITA/OXGR1 on mucus may explain the observation of a lack of significant change in mucin 5b and β-defensin 1 in the airway’s surface liquid.

Collectively, these results provide in vivo evidence to support the role of Oxgr1 and IRG1/ACOD1/ITA in pathogen clearance in the airway (Supplemental Figure 9).

## Discussion

Rapid transcriptional induction of the *IRG1* gene in activated macrophages and the subsequent accumulation of ITA produced by the ACOD1 enzyme is one of the best examples of metabolic reprogramming during activation of the innate immune system. Thus far, most of the reported mechanisms have described the cell-autonomous function of intracellular ITA. Although it has also long been noted that ITA can be secreted from macrophages (4), the function of secreted ITA remains unclear. The present study identifies a function of secreted ITA in signaling through the GPCR protein OXGR1 in respiratory epithelial tissues to promote MCC and innate immunity.

Four lines of evidence collectively establish ITA as a bona fide ligand of OXGR1. First, of the more than 300 GPCRs we screened, OXGR1 was the only one to show robust and reproducible calcium mobilization in response to ITA (Figure 1). Second, ITA activated OXGR1 by interacting with the orthosteric site and induced the canonical intracellular response of G_q_ family G protein activation and receptor endocytosis (Figures 2 and 3). Third, ITA accumulated in mouse airways to sufficiently high levels to activate OXGR1. The possibility of OXGR1 activation by 2 previously reported candidate ligands, α-KG and LTE4, was excluded at least in our experimental system (Supplemental Figures 5 and 6). Fourth, we provide genetic evidence in mouse infection models to support the functional interaction between IRG1/ITA and OXGR1 (Figures 4 and 5). These results show that ITA secreted from activated macrophages is a bona fide paracrine signaling molecule that signals through the GPCR protein OXGR1 in respiratory epithelium to promote MCC and is required for efficient removal of inhaled pathogens during the pulmonary innate immune response (Supplemental Figure 9).

MCC is the primary innate barrier mechanism of the airway and provides a powerful and indispensable defense for the respiratory system, which is continuously exposed to pathogens, particles, and toxic chemicals in inhaled air (14). Defects of mucus clearance make the lung more vulnerable to injury, while excessive mucus production or impaired mucus clearance contributes to multiple respiratory diseases, such as asthma, COPD, and CF (14). P2Y family purinergic GPCR receptors are expressed in airway epithelial cells and sense nucleotides and nucleosides to activate MCC (15), making them potential drug targets for treating respiratory diseases. Denufosol, an inhaled P2Y2 receptor agonist, was shown to improve chloride ion secretion and active MCC, raising the possibility of using airway epithelium agonists to treat patients with CF (43). Since P2Y2 is also expressed in lung-resident immune cells, targeting P2Y2 may have an unfavorable proinflammatory effect (44). Previous studies have shown the robust antiinflammatory effect of ITA in the lung (10, 22), but it may also fuel biofilm production by inhaled pathogens (18, 19). Our finding that ITA/OXGR1 signaling could boost MCC of inhaled pathogens not only reveals a mechanism explaining how the barrier function of the airway is upregulated by innate immune cells during infection, but also identifies OXGR1 as a potential target for developing a synthetic ITA mimic compound to treat mucus clearance defects.

In addition to lung MCC, the ITA/OXGR1 signaling pathway may also operate in tissues where OXGR1 is expressed under other biological and pathological conditions. For instance, Oxgr1 is expressed in renal intercalated cells and regulates bicarbonate secretion to maintain the acid-base balance of body fluids , which is triggered by α-KG in crude urine (31). Inflammatory macrophages in the kidney contribute to salt-sensitive hypertension (45). It will be interesting to determine whether ITA/OXGR1 signaling participates in the regulation of electrolyte homeostasis in the kidney during inflammation. In addition, Oxgr1 is also highly expressed in the trophoblast, and Irg1 is expressed in macrophages in the uterine endometrium during implantation (46). Whether ITA/OXGR1 signaling controls the maternal immune system to facilitate implantation and avoid fetus rejection merits further investigation.

## Methods

### Plasmids, siRNAs, and mouse strain.

The PRESTO-Tango library (Addgene kit no. 1000000068) was a gift from Bryan Roth (University of North Carolina at Chapel Hill, Chapel Hill, North Carolina, USA) (47). Plasmids encoding for human GPCRs were constructed by subcloning the ORFs from PRESTO-Tango to remove the C-terminus reporter sequence. pGloSensor-22F was purchased from Promega. The plasmid for knocking out human OXGR1 was constructed by ligation of sgRNA sequences into pLentiCRISPRv2 (sgOXGR1-1: CGTGGGATTTCCAGGCAATG; sgOXGR1-2: CTAGACTATTTAGCAAATGC). All other plasmids were purchased from Addgene. The siRNA target G_q/11_ (siGNAQ: GACACCGAGAATATCCGCTTT; siGNA11: GCTCAAGATCCTCTACAAGTA) and β-arrestins (siARRB1/2: AAACCTGCGCCTTCCGCTATG siARRB1: AAAGCCTTCTGCGCGGAGAAT; siARRB2: AAGGACCGCAAAGTGTTTGTG) was synthesized by GenePharma. *Oxgr1^–/–^* mice were a gift of Gang Shu (South China Agricultural University, Guangzhou, China) (48). *Irg1^–/–^* mice (JAX, stock no. 029340) were purchased from The Jackson Laboratory. All mice were on a C57BL/6J background.

### Tissue culturing, transfection, and viral transduction.

HEK293 and NIH3T3 cells were purchased from the American Type Culture Collection (ATCC) and cultured in DMEM supplemented with 10% FBS (EXCELL), penicillin, and streptomycin. Primary human bronchial epithelial cells were purchased from Yuchun Biology and maintained in collagen-coated dishes and PneumaCult-Ex Plus medium (STEMCELL Technologies).

For ALI culturing of mouse trachea respiratory epithelium cells, trachea respiratory epithelium was isolated by overnight digestion of 0.15% pronase and cultured in a Transwell insert (0.4 μm pore size, Corning) with proliferation medium (DMEM/F12 supplemented with 10% FBS, bovine pituitary extract, cholera toxin, insulin/transferrin, retinoic acid, and mouse EGF [mEGF]) for approximately 7 days. When they reached full confluence, the cells were differentiated by exposure to air and cultured in a serum-free medium for 20 days. ALI culturing of primary human bronchial epithelium was performed following the manufacturer’s manual (STEMCELL Technologies). Briefly, cells were maintained in PneumaCult-Ex Plus medium on a Transwell insert. Medium on the lateral side was switched to PneumaCult-ALI medium. The apical side of the Transwell membrane was then carefully washed with prewarmed Dulbecco’s PBS (DPBS). Cells were differentiated by exposure to air for 3 weeks. The medium was changed every 2 days.

For BMDM differentiation, bone marrow from the tibia and femur was harvested by flushing the medullary cavity with PBS. The cell suspension was centrifuged at 400*g* for 10 minutes, and the pellets were resuspended in DMEM culture medium supplemented with macrophage CSF (mCSF) (50 ng/μL) and 10% FBS. Cells were cultured and differentiated for 7 days. BMDMs were stimulated with 10 ng/mL LPS for the indicated durations (Figure 1, B–D).

Transfection of plasmids and siRNAs was performed using PolyJet (SinaGen) or Lipofectamine 2000 (Invitrogen, Thermo Fisher Scientific) following the manufacturers’ instructions. For viral transduction, recombinant lentivirus was produced by cotransfection of the packaging plasmids psPAX2 and pMD2G and lentivirus vectors into HEK293T. Cells were infected with the lentivirus for 3 days followed by selection with puromycin for 2 days.

### Aequorin assay, Fluo-4AM assay, and GloSensor assay.

For the aequorin assay, HEK293 cells were transfected with aequorin plasmids (Addgene no. 78734), Gα16 plasmid, and GPCR plasmids. Thirty-six hours after transfection, cells were maintained in serum-free medium for 12 hours and then loaded with 5 μM coelenterazine for 3 hours. Cells were trypsinized and resuspended at a density of 5 × 10^5^ cells/mL in HBSS supplemented with 10 mM HEPES and 0.5% BSA. Cell suspensions of 100 μL were aliquoted into 96-well plates. Ligands (100 μL, diluted to 1.25 mM in HBSS buffer, with a final concentration of 0.625 mM) were injected into plates, and luminance was recorded for 30 seconds using the BioTek Synergy H1 plate reader.

For the Fluo-4AM assay, OXGR1-expressing cells were loaded with 2 μM Fluo-4AM for 30 minutes at 37°C. Excess Fluo-4AM was then washed off with HBSS buffer. After incubation at 37°C for another 10 minutes, cells were transferred onto a 96-well plate. Ligands (α-KG or ITA) were diluted to the indicated concentrations by HBSS buffer supplemented with 30 mM HEPES and injected into the cell suspensions. Fluorescence (488 nm/520 nm) recording was started immediately after injection and recorded every 25 milliseconds for 10 seconds.

For the GloSensor assay, HEK293 cells stably expressing pGloSensor-22F were transfected with GPCR plasmids. Thirty-six hours after transfection, cells were trypsinized and resuspended at a density of 2.5 × 10^5^ cells/mL in HBSS supplemented with 10 mM HEPES, 0.5% BSA, 2% GloSensor cAMP Reagent stock solution (Promega), and 300 μM IBMX, and loaded with GloSensor substrates for 2 hours. Cell suspensions (100 μL) were aliquoted onto 96-well plates. Cells were treated with 0.5 mM of the indicated ligand or 1 μM forskolin, and luminance was recorded.

### Western blot analysis.

HEK293 cells were transiently transfected with plasmids expressing empty vector or FLAG-tagged OXGR1 using PolyJet reagent. The medium was switched to serum-free DMEM 36 hours after transfection. After serum starvation for another 12 hours, cells were stimulated with the indicated ligand and lysed in Laemmli buffer. Protein lysate was separated on standard SDS-PAGE, transferred onto a PVDF membrane, and then subjected to immunoblotting with the following antibodies: anti–p-ERK (catalog 4370, Cell Signaling Technology); anti-Flag (catalog 14793, Cell Signaling Technology); anti-IRG1/anti-ACOD1 (catalog 19857, Cell Signaling Technology); anti-Myc (catalog 2278, Cell Signaling Technology); anti-ERK (catalog 67170-1-Ig, Proteintech); and anti-actin (catalog A00702, GenScript).

### Immunofluorescence microscopy.

For endocytosis assays, HEK293 cells stably expressing FLAG-OXGR1 were chilled on ice in DMEM medium supplemented with 30 mM HEPES for 10 minutes. Cells were incubated with anti-FLAG antibody (catalog 14793, Cell Signaling Technology) on ice for 60 minutes. Unbound antibody was then washed off with prechilled HBSS buffer. Endocytosis was induced by incubating cells with the indicated ligand (α-KG or ITA) in HBSS buffer at 37°C for 10 minutes. After ligand stimulation, cells were fixed and permeabilized. Subcellular localization of FLAG-tagged OXGR1 was detected using FITC-donkey anti-rabbit antibody (Invitrogen, Thermo Fisher Scientific), and nuclei were stained with DAPI (MilliporeSigma). Immunofluorescence images were captured with a Leica SP8 laser-scanning confocal microscope.

For the stress fiber assay, NIH3T3 cells stably expressing OXGR1 were treated with 500 μM ITA or 10 μM lysophosphatidic acid (LPA) for 30 minutes. Cells were fixed with 4% paraformaldehyde and permeabilized with 0.1% Triton X-100. Stress fibers were stained with FITC-phalloidin (Invitrogen, Thermo Fisher Scientific). Nuclei were stained with DAPI. Immunofluorescence images were captured with a Leica SP8 laser-scanning confocal microscope.

### Calcium imaging.

For calcium imaging of human primary bronchial epithelial cells, cells were infected with 2 CRISPR lentiviruses targeting OXGR1 and cultured for 2 days before calcium imaging. For calcium imaging of primary mouse cells, freshly isolated mouse tracheas were cut into rings, placed onto collagen-coated dishes, and cultured in DMEM supplemented with 10% FBS. Cells were loaded with 5 μM Fluo-4AM for 30 minutes in HBSS buffer, washed twice, and incubated for another 15 minutes for fully deesterification of Fluo-4AM. Cells loaded with Fluo-4AM were monitored with the BioTek Cytation 5 Cell imaging system every 300 ms for a total of 1.5 minutes after ligand administration. Fluorescence was quantified with ImageJ software (NIH).

### Measurement of particle transport.

For the measurement of bead movement in ALI culture, the apical side of the ALI culture inserts was washed with PBS 5 times to fully remove mucus. The inserts were transferred to HBSS buffer supplemented with 30 mM HEPES and the indicated ligand, and 20 μL bath buffer containing red fluorescent beads was added to the apical side. After 15 minutes of equilibration, bead movement was monitored with the BioTek Cytation 5 cell imaging system.

Tracheas were harvested from mice and cut longitudinally through the middle of the trachealis muscle to expose the lumen of the trachea. Tracheas were then transferred into HBSS buffer and equilibrated for 30 minutes. With the lumen facing down, trachea tissues were transferred into 2 mL HBSS buffer supplemented with 0.5% methylcellulose, red fluorescent beads, 30 mM HEPES, and the indicated ligand. Bead flow was monitored with the BioTek Cytation 5 cell imaging system. Twenty individual beads were tracked from different fields of view in each condition, and the speed of each bead was calculated by dividing the length of its trajectory.

### Histological analysis of nasal cavity.

Harvested mouse skulls were fixed in 4% paraformaldehyde overnight and decalcified with 12% EDTA for 2 weeks. After full decalcification, the snoots were trimmed in between the incisor teeth and the first molar teeth. Trimmed tissues were embedded with paraffin, and sections were taken from the most proximal aspect of the nasal cavity. Mucin-containing epithelial cells distributed in the basement membrane of the nasal cavity floor and septum were visualized by Alcian blue/PAS staining and manually counted.

### Mouse intranasal infection.

A 1 × 10^7^ log-phase PAO1 strain in 40 μL PBS was used for infection of the mice. Mice were gently restrained by the scruff at a 45° angle. PBS with the indicated strains was delivered to the nares. PBS alone or with 1 mM ITA was codelivered along with PAO1. Mouse mouths were covered during the whole procedure to ensure complete inhalation into the nose and the lower respiratory tract. BAL and lung tissue were collected 12 hours of infection and plated in cetyl trimethyl ammonium bromide (CTAB) selection agar. The numbers of CFU were counted after 18 hours of culture.

### Quantification of metabolite concentrations.

The concentrations of urea in plasma and BAL were determined using a urea assay kit (Solarbio). For ITA determination, samples were extracted by 80% (vol/vol) chilled (–80°C) methanol and analyzed with an ultra-high-performance liquid chromatograph (Acquity UPLC I-Class, Waters) coupled to a triple quadrupole mass spectrometer (Xevo TQ-XS, Waters). For α-KG determination, samples were lyophilized and derived by methoxyamine (MilliporeSigma) and *N*-methyl-*N*-[tert-butyldimethylsilyl] trifluoroacetamide (MilliporeSigma) in pyridine (MilliporeSigma). The derivatized sample was injected into an Agilent 7890A-5975C GC-MS system with an HP-5MS column.

### Statistics.

Statistical analyses were performed using GraphPad Prism 8.0 (GraphPad Software). A 2-tailed Student’s *t* test was used for comparisons between 2 groups. A 1-way ANOVA with Tukey’s multiple-comparison test was used for comparisons of more than 2 groups. Data are presented as the mean ± SEM. *P* values of less than 0.05 were considered statistically significant.

### Study approval.

All animal experiments were approved and supervised by the Animal Research Committee (ARC) of Fudan University (Shanghai, China) with the appropriate ethics regulations.

## Supplementary Material

Supplemental data

## Version 1 03/15/2023

Electronic publication
