# Supplementary material for: The immunometabolite itaconate stimulates OXGR1 to promote mucociliary clearance during the pulmonary innate immune response
Source: J Clin Invest. 2023 Mar 15;133(6):e160463. doi: 10.1172/JCI160463 (PMC10014103; doi:10.1172/JCI160463)
Supplement: Supplemental data [file jci-133-160463-s086.pdf]

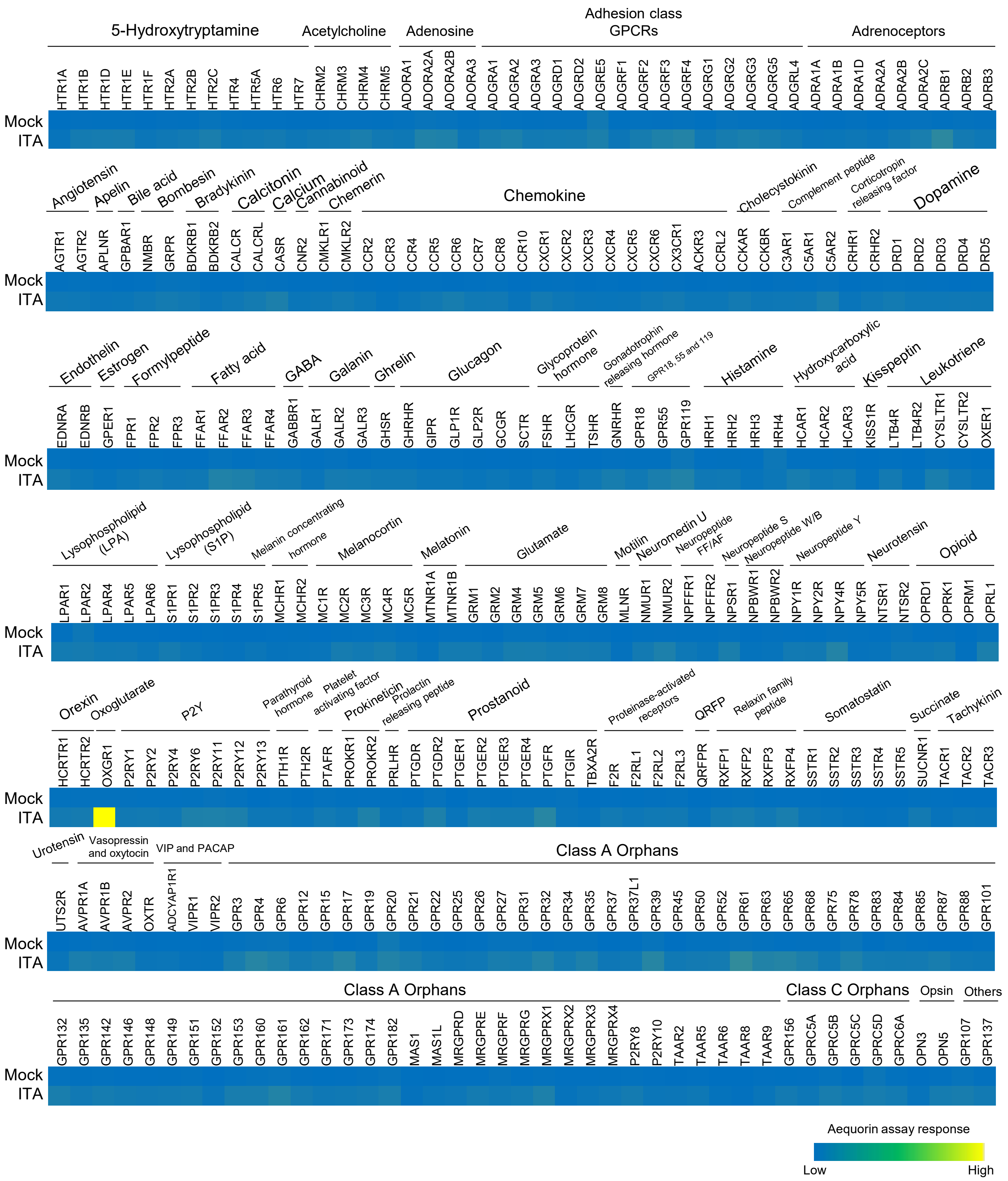

Supplementary Figure 1. A screen of GPCR receptors discovers OXGR1 as an ITA receptor

The aequorin assay response of cells overexpressing indicated GPCR was quantified and shown as a heatmap. The classification of GPCR is according to the International Union of Basic and Clinical Pharmacology (IUPHAR).

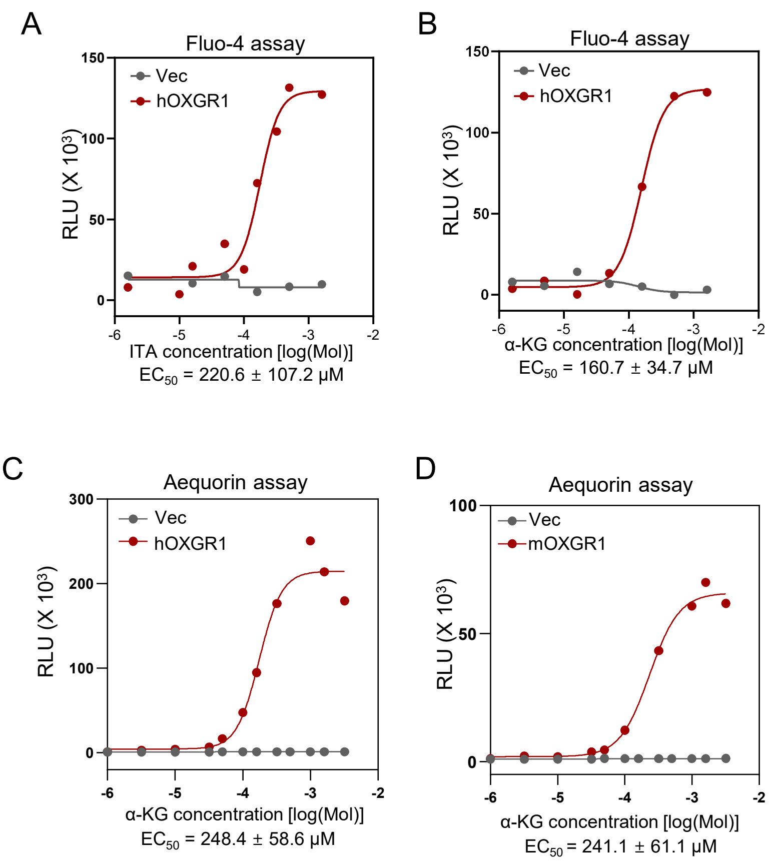

Supplementary Figure 2. ITA acts as an agonist of OXGR1 to increase cytosolic calcium

A, B. The response of human OXGR1 to ITA or α-KG in HEK293 cells, as determined by the Fluo-4 assay. **C, D.** The response of human and mouse OXGR1 to α-KG in HEK293 cells, as determined by the aequorin assay.

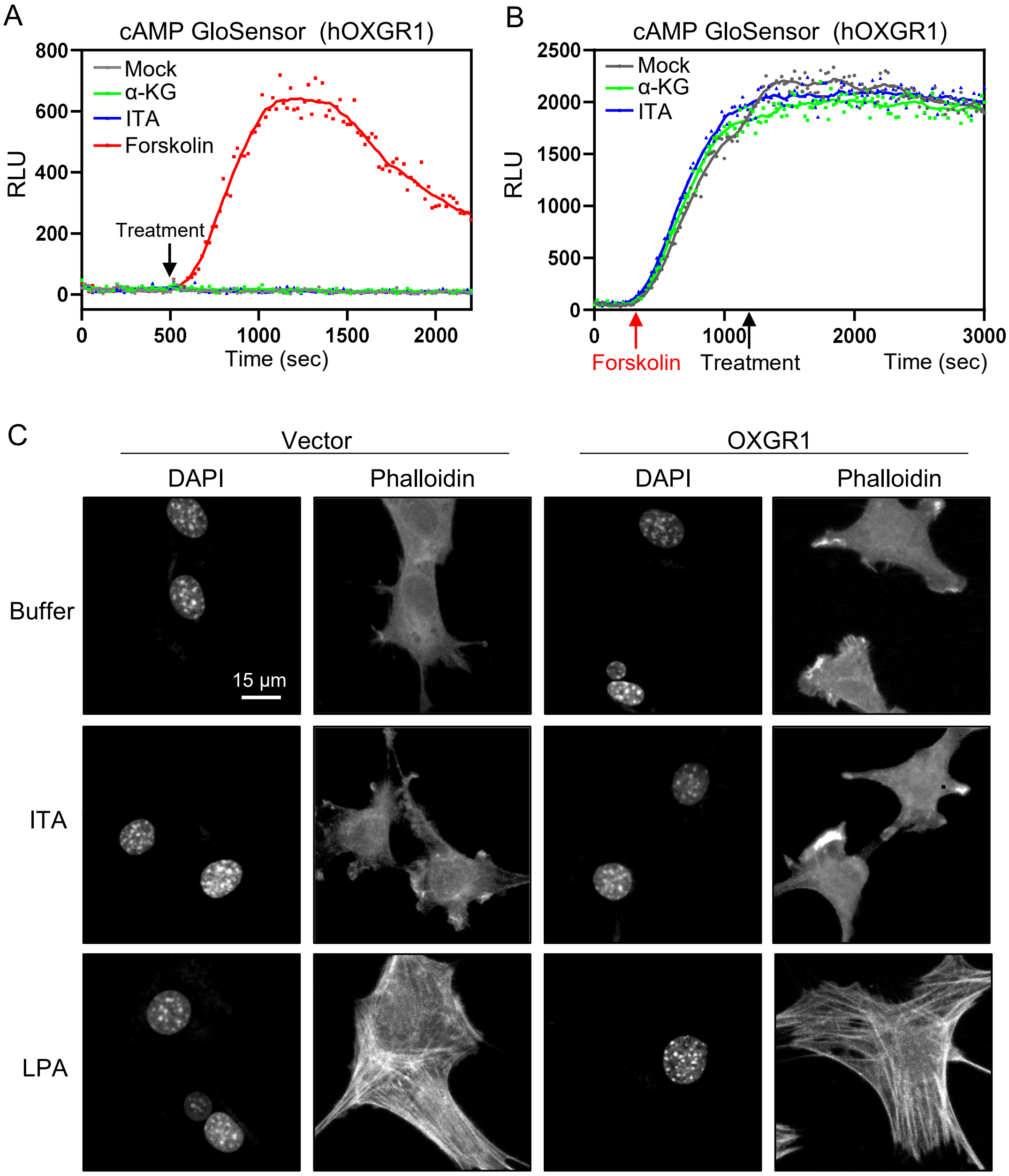

Supplementary Figure 3. ITA-OXGR1 does not couple to G_s, G_i, or $G_{12/13}$

A, B. cAMP was measured by cAMP GloSensor assay in HEK293 cells expressing human OXGR1. Forskolin was included as a positive control. **C.** NIH3T3 cells with or without overexpression of human OXGR1 were treated with indicated ligands. The stress fiber was stained with FITC-conjugated phalloidin, and the nucleus was stained by DAPI. LPA was included as a positive control to induce stress fiber formation. Scale bar,15 μm.

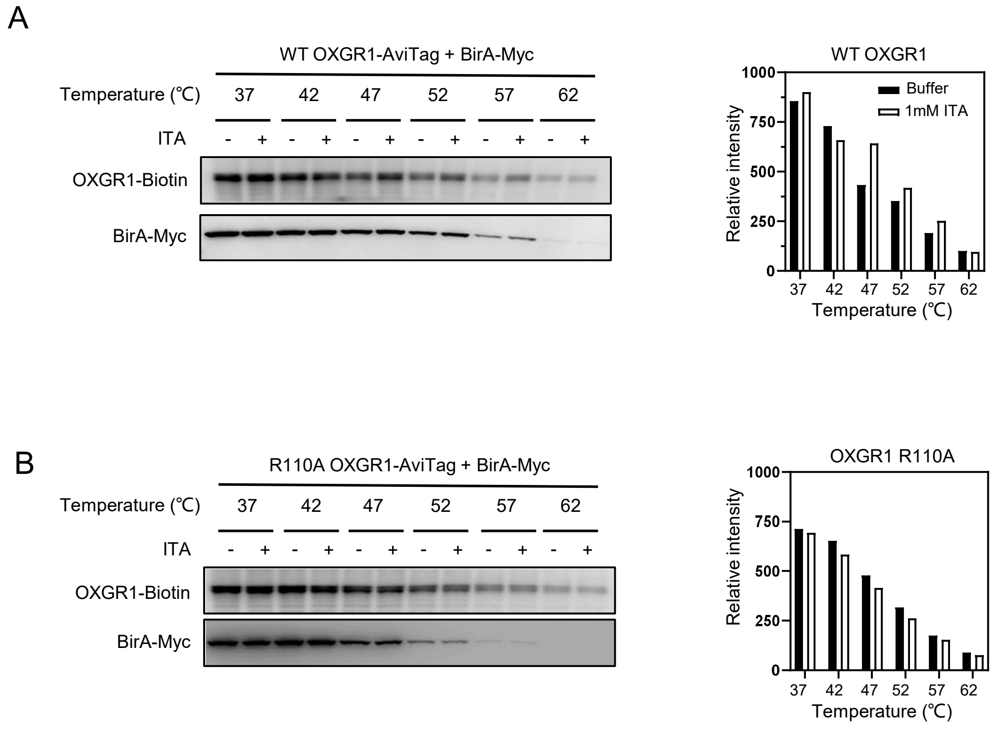

Supplementary Figure 4. ITA increases the thermostability of OXGR1

Cells expressing Myc-tagged BirA along with AviTag-tagged wildtype (A) or R110A mutant (B) OXGR1 were treated with 1 μM biotin for 16 hours in serum-free medium to biotinylate OXGR1 in native conformation. Cells were treated with or without 1mM ITA, lysed, and incubated under different temperatures (from 37 to 62℃) for 3 minutes. Denatured protein was removed by centrifuge. Biotinylated OXGR1 was determined by Western blot and quantified.

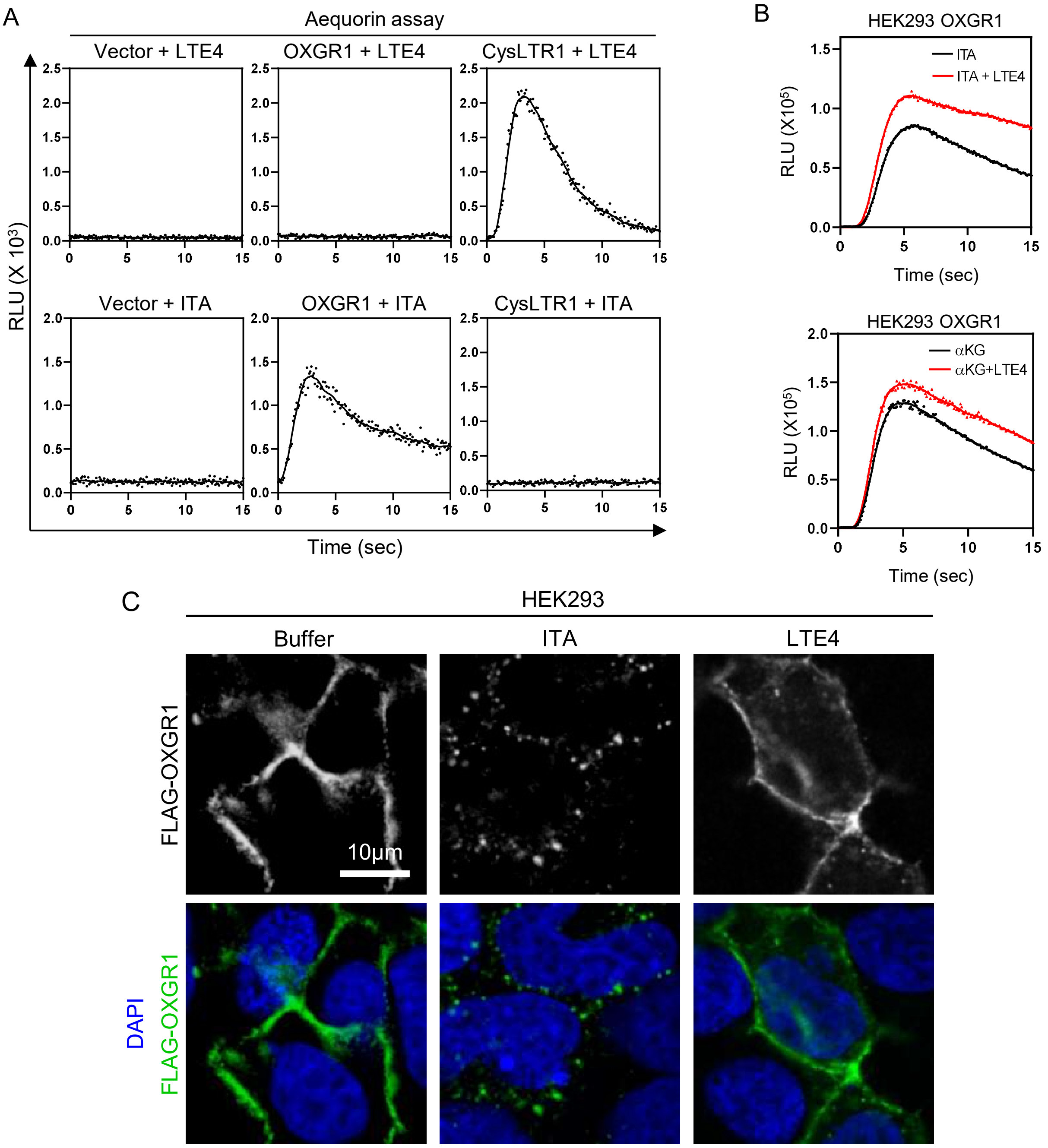

Supplementary Figure 5. LTE4 is not a direct agonist of OXGR1

A. Aequorin assay showing calcium mobilization in HEK293 cells with overexpression of human OXGR1 or CysLTR1 after stimulation by LTE4 and ITA. **B.** Aequorin assay showing calcium mobilization in HEK293 cell with overexpression of human OXGR1 after stimulation by ITA/α-KG in combination with LTE4**.** **C.** Endocytosis of Flag-tagged OXGR1 induced by ITA or LTE4 treatment was detected by anti-Flag antibody. Scale bar,10μm.

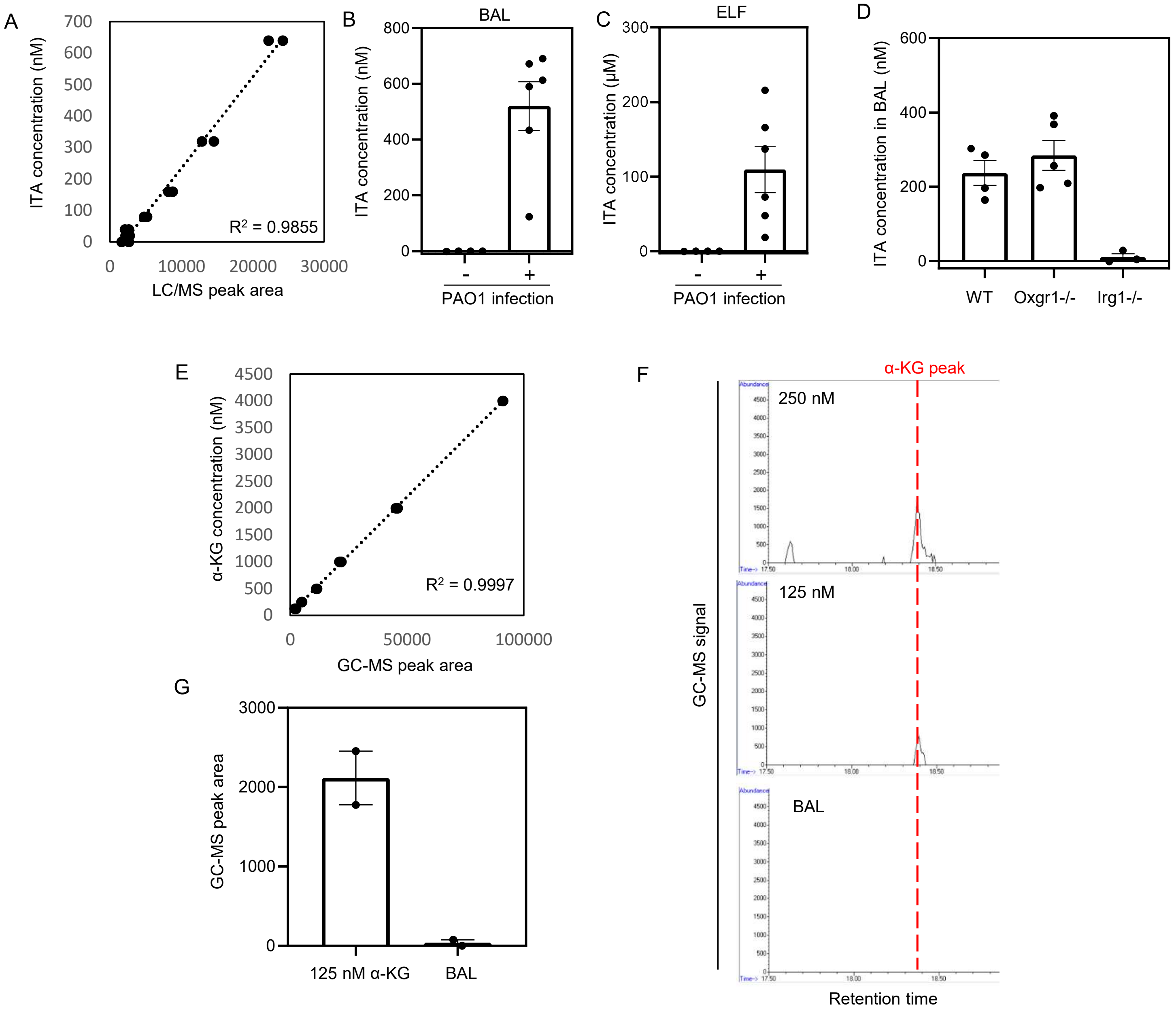

Supplementary Figure 6. ITA and α-KG concentrations in BAL from mice after *P. aeruginosa* infection

A. Standard curve of ITA determined by LC-MS. **B.** ITA concentration in 3 mL BAL of wildtype mice treated with or without PAO1 at 12 hours post infection (n=4-6). **C.** The concentration of ITA in undiluted epithelial lining fluid (ELF) was calculated from BAL by using urea concentration as a marker of dilution. **D.** ITA concentration in 3 mL BAL of mice with indicated genotypes 12 hours post PAO1 infection (n=3-5). **E.** Standard curve of α-KG determined by GC-MS. **F.** GC-MS chromatogram of α-KG standards (250 nM, 125 nM) and a concentrated BAL sample. The red line indicates the α-KG peak. Peak areas were quantified in panel **G**.

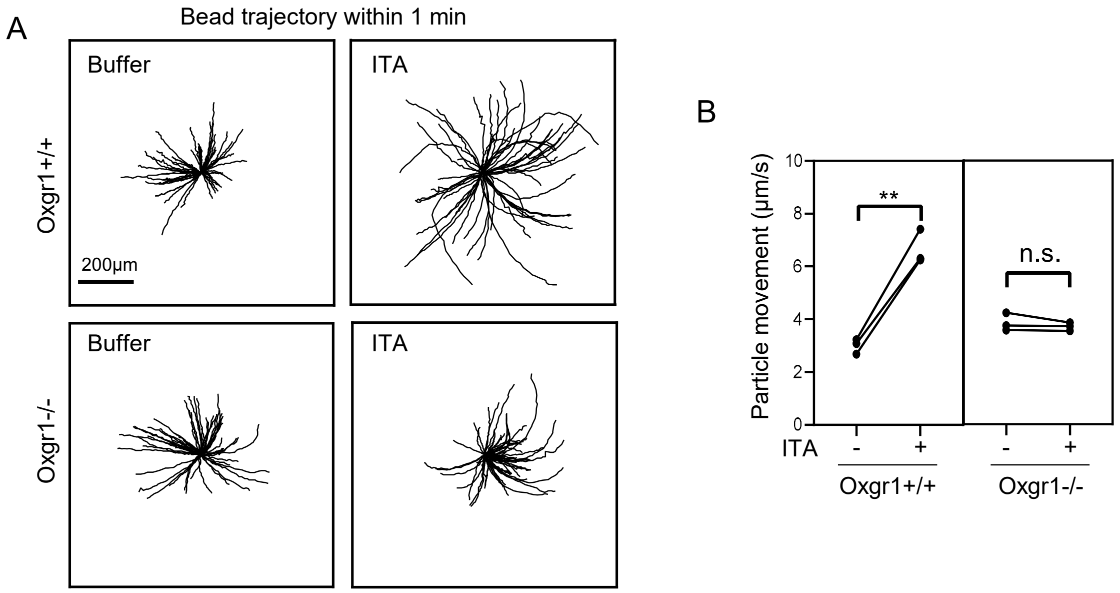

Supplementary Figure 7. ITA acts as an agonist of OXGR1 to active particle transport

A. The trajectory of 60 individual beads within 1 min in mouse trachea epithelial cell ALI culture with or without 500 μM ITA treatment. Scale bar, 200 μm. **B.** Bead movement speed was quantified. Each dot represents an independent biological replicate. Significance is determined by paired t-test. **indicates $p<0.01$; n.s., no significance; error bar, SEM.

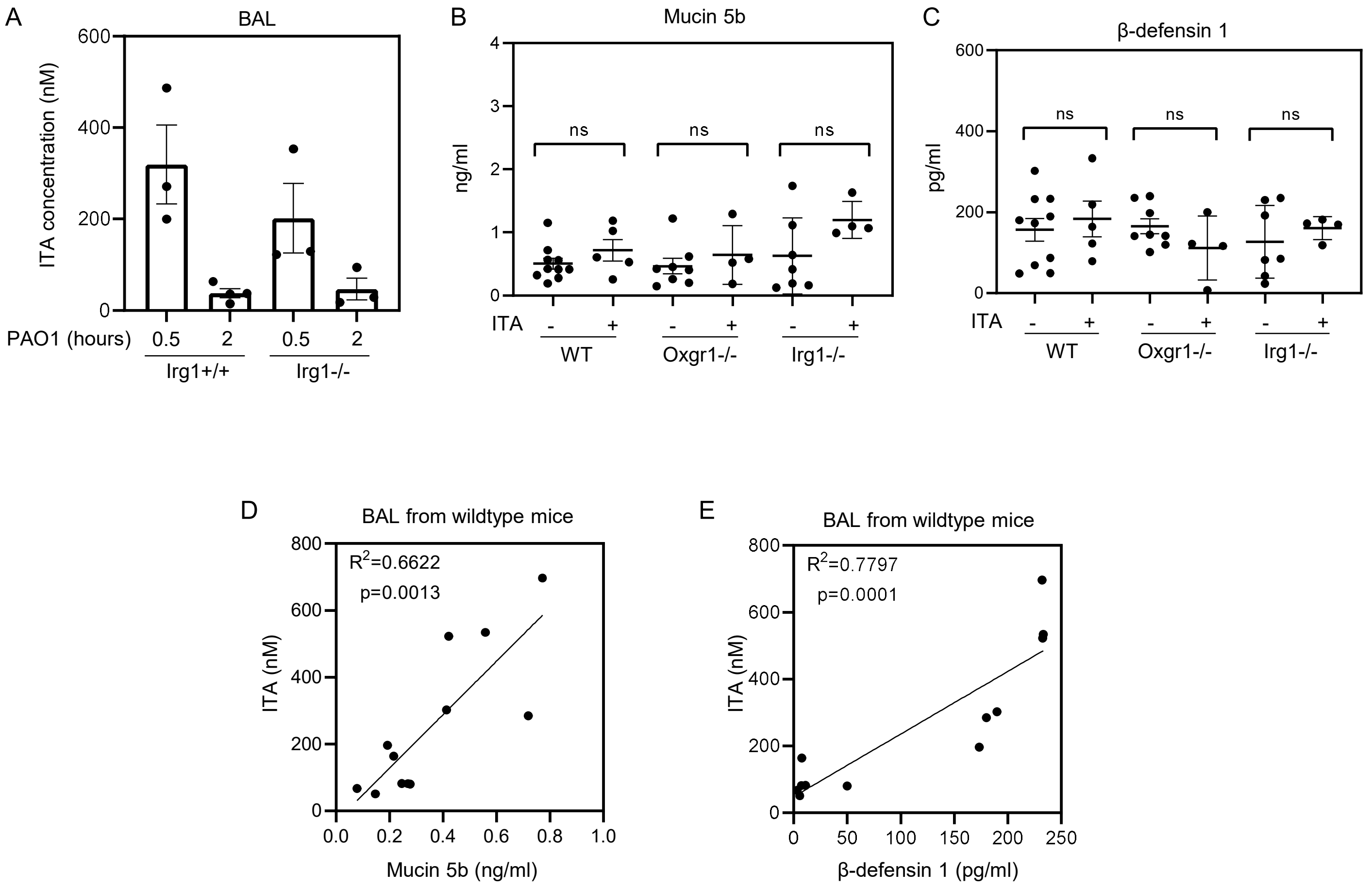

Supplementary Figure 8. ITA level is positively correlated with Mucin 5b or β-defensin 1 concentration in BAL from mice after *P. aeruginosa* infection

A. ITA concentration in BAL was determined by LC-MS after intranasal delivery of 20 μL saline containing PAO1 and 1mM ITA (n=3-4). **B-C.** Comparison of Mucin 5b (B) and β-defensin1 (C) concentration in BAL of *P. aeruginosa* infected mice with indicated genotypes (n=4-10). Significance is determined by paired t-test. n.s., no significance. **D-E.** Correlation analysis of ITA concentration and Mucin 5b (D) or β-defensin1 concentration (E) in BAL of *P. aeruginosa* infected WT mice.

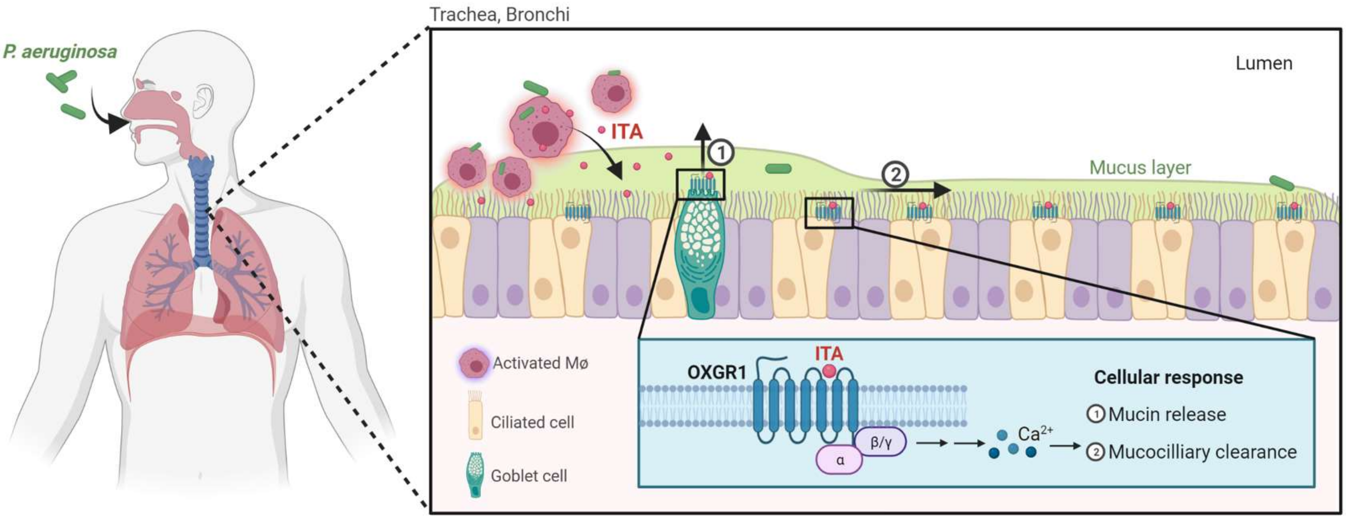

Supplementary Figure 9. Schematic model of ITA-Oxgr1 signaling in mucociliary clearance

During airway infection, activated macrophages produce and secret ITA, which acts through OXGR1 expressed in respiratory epithelial cells to promote mucociliary clearance.
